# Green Phosphors Based on 9,10-bis((4-((3,7-dimethyloctyl)oxy) phenyl) ethynyl) Anthracene for LED

**DOI:** 10.3390/mi10100703

**Published:** 2019-10-15

**Authors:** Xuefeng Ren, Hai Song, Jing Xiao, Hui-Juan Yu, Chi-Fang Peng, Guang Shao

**Affiliations:** 1 College of Chemistry and Chemical Engineering, Hexi University, Zhangye 734000, China; rxfwhy@sina.com (X.R.); songh-@163.com (H.S.); 2 Key Laboratory of Hexi Corridor Resources Utilization of Gansu, Hexi University, Zhangye 734000, China; 3 School of Chemistry, Sun Yat-Sen University, Guangzhou 510275, China; xiaoj69@mail.sysu.edu.cn; 4 Shenzhen Research Institute, Sun Yat-sen University, Shenzhen 518057, China; 5 School of Food Science and Technology, Jiangnan University, Wuxi 214122, China; pcf@jiangnan.edu.cn

**Keywords:** phosphors, luminescence, chemical synthesis, bathochromic shift

## Abstract

An anthracene aromatic unit was introduced into the phenylethynyl structure by a rigid acetylene linkage at the C-9 and C-10 positions via Sonogashira coupling reactions, resulting in a planar and straight-backbone molecule (9,10-bis((4-((3,7-dimethyloctyl)oxy) phenyl) ethynyl) anthracene) (BPEA). Thermogravimetric analysis demonstrated the good thermal stability of the BPEA. Photoluminescence analysis showed that a suitable expanded π-conjugation in the BPEA made its excitation band extend into the visible region, and an intense green emission was observed under blue-light excitation. A bright green light-emitting diode with an efficiency of 18.22 lm/w was fabricated by coating the organic phosphor onto a 460 nm-emitting InGaN chip. All the results indicate that BPEA is a useful green-emitting material which is efficiently excited by blue light, and therefore, that it could be applied in many fields without UV radiation.

## 1. Introduction

Light-emitting diode (LED) lamps using phosphor conversion of 350–480 nm LED radiation have drawn much attention, and are notably expected to replace traditional illumination sources due to their superior features such as low power consumption, long lifetime, high efficiency, small volume, and low maintenance [1,2,3]. The InGaN-based white light-emitting diode (WLED) is a new kind of solid-state illumination technology, the efficiency of which has already surpassed that of traditional incandescent bulbs and fluorescent lamps [4,5,6,7,8,9,10,11]. WLEDs can be achieved by the incorporation of tricolor phosphors (blue, green, and red) with near-UV-InGaN chips or phosphors (green and red) with blue InGaN chips. Phosphor materials play a very important role in WLEDs [4,5,6,7,8]. Although near-UV-InGaN chips can offer higher energy to pump the phosphors, they also present low photochemical stability under ultraviolet UV irradiation [9,10]. As a result, it is necessary to develop phosphors which may be excited by blue InGaN LED chips that have high levels of efficiency and small thermal quenching. However, the search for phosphors with a suitable expanded π-system that can be excited by blue InGaN LED chips is ongoing [11].

Organic materials composed of aromatic groups conjugated through acetylene linkages have been widely used as optoelectronic materials due to the characteristic features emerging from their skeletal persistency and labile π-electron systems [12,13,14]. It has been reported that some phenyl acetylene molecules could show unique intermolecular aggregation through coplanar interactions, leading to efficient charge transport, fast energy transfer, and excellent luminescence properties, both in solution and in a solid state [15,16]. Furthermore, their highest occupied molecular orbital (HOMO)-lowest unoccupied molecular orbital (LUMO) energy gaps and emissive properties can be controlled by tuning their effective conjugation lengths [17,18]. It is well known that the anthracene has a regular planar π-system, and that it may be highly capable of π-stacking. It is anticipated that such a strong interaction will facilitate the construction of planar and straight-backbone structures and further extend the conjugation length of the phenyl acetylene chromophores. As a result, the excitation bands of chromophores could be extended into the visible region [19,20]. In recent years, many organic materials containing an anthracene unit have been extensively studied and developed as blue-light-emitting materials in OLEDs because of their excellent photoluminescence (PL) and electroluminescence (EL) properties [21]. Among the derivatives containing an anthracene core, 9,10-diphenylanthracene is an attractive aromatic unit due to its high fluorescence in its solid state [22,23,24].

In this article, an anthracene fragment was connected to a phenylethynyl structure by a rigid acetylene linkage at the C-9 and C-10 positions, resulting in an efficient π-conjugation molecule. The bulky 3,7-dimethyloctyloxy substituent was introduced to circumvent the problem of insolubility and aggregation-caused quench originating from the rigid anthracene fragment. A photoluminescence analysis showed that this molecular modification significantly extended the excitation band of 9,10-bis((4-((3,7-dimethyloctyl)oxy) phenyl) ethynyl) anthracene (BPEA) into the blue region, which exhibited an intense green emission under blue-light excitation. Finally, a bright green-emitting LED was fabricated by coating the BPEA onto a 460 nm-emitting InGaN chip.

## 2. Experimental

### 2.1. General

All reactions were carried out under an atmosphere of argon with freshly-distilled solvents, unless otherwise noted. Toluene, diisopropylamine, and methylene chloride were distilled from calcium hydride. Silica gel was used for column chromatography. The NMR spectra were recorded at 25 °C on JEOL Lambda 300 and 500 instruments (Tokyo, Japan) and calibrated with tetramethylsilane as an internal reference. An elemental analysis was performed on a Perkin-Elmer PE 2400 instrument (Waltham, MA, USA). The electron impact ionization mass spectra (EI-MS) were recorded on a Thermo DSQ mass spectrometer (Waltham, MA, USA) by direct inlet at room temperature. IR spectra were recorded as potassium bromide pellets on an IR-Nicolet Avatrar 330 spectrometer (Thermo Fisher Scientific, Waltham, MA, USA) at room temperature. A UV-visible absorption spectrum was recorded on UV 2550 (Shimadzu, Kyoto, Japan) at room temperature. The excitation and emission spectra were recorded on a RF5301 (Shimadzu, Kyoto, Japan, slit width 1.5 nm) at room temperature. The emission spectra of the fabricated LEDs were measured with an Everfine PMS-50 PLUS UV–vis–near IR spectrophotocolorimeter (Hangzhou, China) at room temperature. A thermogravimetric analysis (TGA) was carried out from 35 °C to 930 °C with a heating speed of 10.0 K/min in a N_2_ atmosphere on a Netzsch thermogravimetric analyzer (STA449F3, NETZSCH, Selb, Germany).

The quantum yield in solution was measured at room temperature using an integrated sphere system (External Quantum Efficiency Measurement System C9920-12, Hamamatsu Photonics, Hamamatsu, Japan). The quantum yield in the solid state was measured at room temperature using an Everfine PMS-50 PLUS UV–vis–near IR spectrophotocolorimeter integrating sphere. Differential scanning calorimetry (DSC) measurements were recorded with a differential scanning calorimeter (LIFM 200F3, NETZSCH, Selb, Germany).

### 2.2. Synthesis

**Scheme 1:**
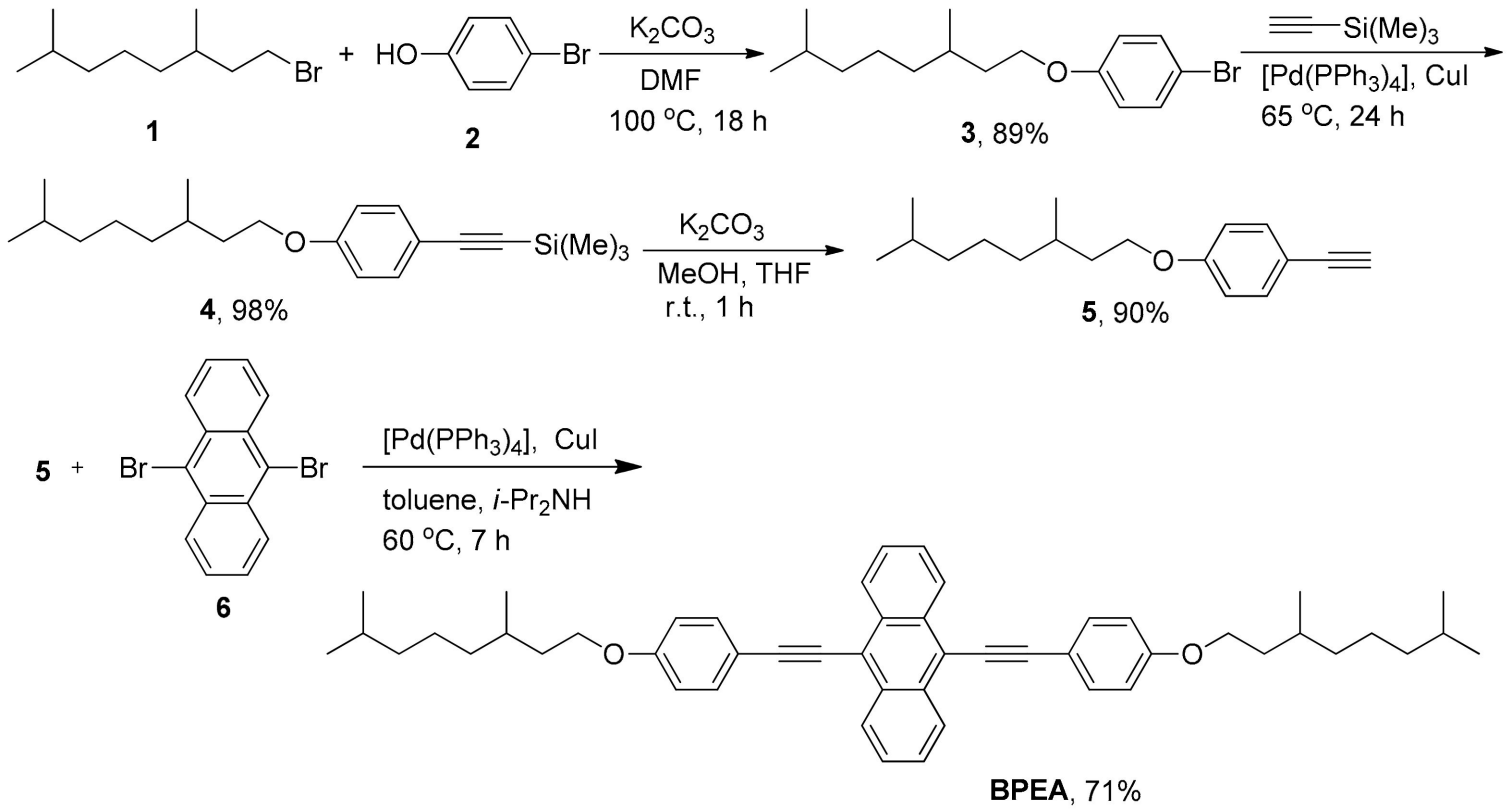
Synthesis of BPEA.

The synthetic procedures of BPEA were straightforward, as shown in Scheme 1. In order to circumvent the problem of insolubility and aggregation-caused quenching, the aromatic compound containing an anthracene fragment was designed to bear a bulky 3,7-dimethyloctyloxy substituent. Although the syntheses of compounds 3–5 have been reported [25], our synthetic procedures are also provided (Scheme 1). BPEA was synthesized by Sonogashira coupling of 9,10-dibromoanthracenewith terminal ethyne 5, which was obtained by desilylation of compound 4 with K_2_CO_3_ in MeOH/THF.

#### 2.2.1. Synthesis of Compound 3

First, 1-Bromo-3,7-dimethyloctanre (4.20 g, 19.00 mmol) was added to a solution of 4-bromophenol (3.46 g, 20.00 mmol) and K_2_CO_3_ (13.82 g, 100.00 mmol) in 60 mL DMF. After stirring at 100 °C for 18 h, the reaction mixture was cooled to room temperature. After the addition of water, the organic layer was extracted with ethyl acetate, washed with brine, and dried over magnesium sulfate. The solvents were evaporated and the residue was subjected to column chromatography to yield the desired product (5.30 g, 89%). N.B. ^1^H NMR (300 MHz, CDCl_3_): *δ* in ppm = 0.86 (d, *J* = 6.6 Hz, 6H), 0.92 (d, *J* = 6.4 Hz, 3H), 1.13–1.32 (m, 6H), 1.50–1.59 (m, 3H), 1.78–1.82 (m, 1H), 3.92–3.97 (m, 2H), 6.77 (d, *J* = 8.9 Hz, 2H), 7.35 (d, *J* = 8.9 Hz, 2H); ^13^C NMR (125 MHz, CDCl_3_): *δ* in ppm = 19.6, 22.5, 22.6, 24.6, 27.9, 29.8, 36.0, 37.2, 39.2, 66.5, 112.5, 116.2, 132.1, 158.2.

#### 2.2.2. Synthesis of Compound 4

(Trimethylsilyl) acetylene (2.50 g, 3.52 mL) was added to a solution of 1-(3,7-dimethyloctyloxy)-4-bromobenzene (5.30 g, 16.92 mmol), [Pd(PPh_3_)_4_] (982.2 mg, 0.85 mmol) and CuI (161.8 mg, 0.85 mmol) in 80 mL toluene and 20 mL diisopropylamine. After stirring at 65 °C for 24 h, the reaction mixture was cooled to room temperature and filtered. The filtrate was poured into aqueous NH_4_Cl and extracted with ethyl acetate. The extract was then washed with brine, dried over magnesium sulfate and filtered. After evaporation, the residue was subjected to column chromatography to yield the desired product in a pure form (5.48 g, 98%). N.B. ^1^H NMR (500 MHz, CDCl_3_): *δ* in ppm = 0.23 (s, 9H), 0.86 (d, *J* = 6.7 Hz, 6H), 0.93 (d, *J* = 6.4 Hz, 3H), 1.14–1.32 (m, 6H), 1.49–1.64 (m, 3H), 1.77–1.83 (m, 1H), 3.93–4.01 (m, 2H), 6.80 (d, *J* = 8.7 Hz, 2H), 7.38 (d, *J* = 8.7 Hz, 2H); ^13^C NMR (125 MHz, CDCl_3_): *δ* in ppm = 0.07, 19.6, 22.5, 22.6, 24.6, 27.9, 29.7, 36.0, 37.2, 39.2, 66.2, 92.1, 105.3, 114.3, 114.9, 133.3, 159.3.

#### 2.2.3. Synthesis of Compound 5

K_2_CO_3_ (20.0 g) was added to a solution of [(4-(3,7-dimethyloctyloxy) phenyl) ethynyl]-trimethylsilane (6.61 g, 20.0 mmol) in a mixture of THF (30 mL) and MeOH (40 mL). The reaction mixture was stirred for 1 h at room temperature. After the addition of water, the organic layer was extracted with ethyl acetate, washed with brine, and dried over magnesium sulfate. The solvents were evaporated and the residue was subjected to column chromatography to yield the desired product (4.65 g, 90%). N.B. ^1^H NMR (300 MHz, CDCl_3_): *δ* in ppm = 0.86 (d, *J* = 6.6 Hz, 6H), 0.93 (d, *J* = 6.4 Hz, 3H), 1.14–1.34 (m, 6H), 1.43–1.62 (m, 3H), 1.76–1.85 (m, 1H), 2.99 (s, 1H), 3.96–4.01 (m, 2H), 6.83 (d, *J* = 8.7 Hz, 2H), 7.41 (d, *J* = 8.7 Hz, 2H); ^13^C NMR (125 MHz, CDCl_3_): *δ* in ppm = 19.6, 22.5, 22.6, 24.6, 27.9, 29.8, 36.0, 37.2, 39.2, 66.3, 75.6, 83.7, 113.8, 114.4, 133.5, 159.5.

#### 2.2.4. Synthesis of BPEA

A 50 mL flask was charged with [4-(3,7-dimethyl)-octyloxy ] phenylacetylene (357 mg, 1.38 mmol), 9, 10-dibromoanthracene (202 mg, 0.60 mmol), [Pd(PPh_3_)_4_] (69 mg, 0.06 mmol), CuI (11 mg, 0.06 mmol), diisopropylamine (2 mL), and toluene (20 mL). After stirring at 60 °C for 7 h, the reaction mixture was cooled to room temperature and filtered. The filtrate was poured into aqueous NH_4_Cl and extracted with CH_2_Cl_2_. The extract was then washed with brine, dried over magnesium sulfate, and filtered. After evaporation, the residue was subjected to column chromatography to yield the desired product, i.e., a brown powder in a pure form (294 mg, 71%). N.B. m.p.: 116–118 °C. ^1^H NMR (500 MHz, CDCl_3_): *δ* in ppm = 0.88 (d, *J* = 6.7 Hz, 12H), 0.97 (d, *J* = 6.4 Hz, 6H), 1.17–1.35 (m, 12H), 1.54–1.70 (m, 6H), 1.85–1.89 (m, 2H), 4.04–4.08 (m, 4H), 6.97 (d, *J* = 8.7 Hz, 4H), 7.61-7.63 (m, 4H), 7.70 (d, *J* = 8.7 Hz, 4H), 8.67–8.69 (m, 4H).^13^C NMR (125 MHz, CDCl_3_): *δ* in ppm = 19.6, 22.6, 22.7, 24.6, 27.9, 29.8, 36.1, 37.2, 39.2, 66.4, 85.3, 102.5, 114.7, 115.3, 118.4, 126.5, 127.3, 131.9, 133.1, 159.5. IR (KBr): *v_max_*= 3422, 3058, 2955, 2924, 2867, 2537, 2188, 1601, 1508, 1463, 1388, 1289, 1250, 1166, 1020, 824, 765 cm^-1^. MS (EI) *m/z* (%) 690 (100) [M+], 691 (41) [M+1], 692 (12) [M+2]. Elemental analysis (%): Calcd for C_50_H_58_O_2_: C, 86.91; H, 8.46. Found: C, 86.69; H, 8.70.

### 2.3. Fabrication of LED

A green-emitting LED was fabricated by combining a ~460 nm-emitting InGaN chip with BPEA as phosphors. Firstly, the powder of BPEA was mixed with a commercially-available silicone gel (6175A and 6175B, mass ratio of 1:1, purchased from Sil-More Industrial Ltd., Taiwan) in a mass ratio of 1:20 (BPEA: Silicone gel); then, the mixture was coated onto a ~460 nm-emitting InGaN chip. The modified InGaN chip was dried in an oven at 150 °C for 1 h. In order to prevent the phosphor from dispersing into the epoxy resin, another silicone gel layer was coated onto the phosphor and dried at 150 °C for another 1 h. Finally, the whole LED lamp was completely encapsulated with a transparent epoxy resin [26].

## 3. Results and Discussion

### 3.1. Thermal Stability

**Figure 1:**
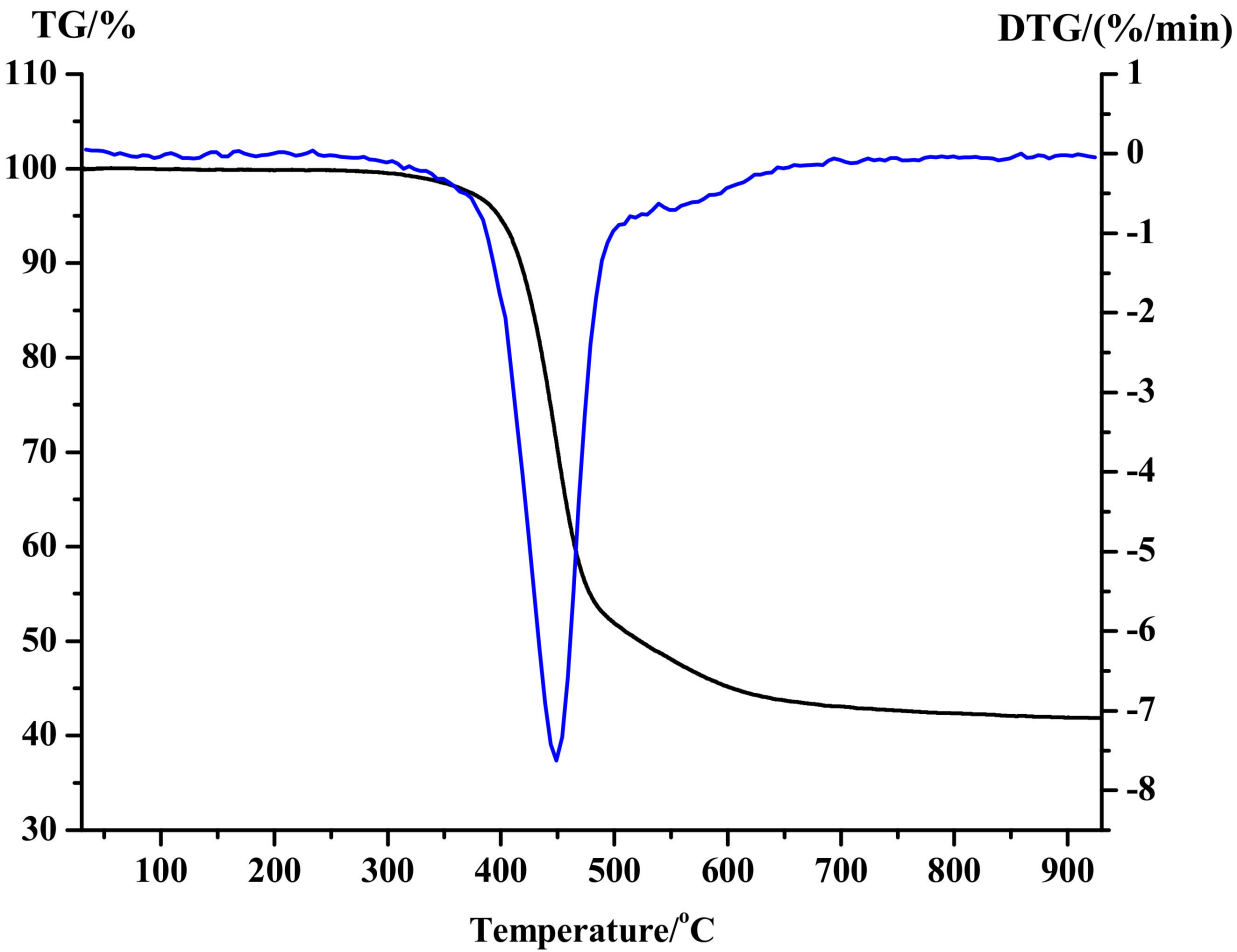
TGA (black) and DTG (blue) of BPEA.

An organic phosphor applied in the fabricated LEDs is required to achieve a high level of thermal stability. A thermogravimetric analysis (TGA) and differential thermogravimetric (DTG) techniques were employed to investigate the thermal stability of the synthesized compound. The decomposition temperature here is defined as the temperature at which 5% weight loss occurs during heating in nitrogen [27]. It was found from the TGA curves presented in Figure 1 that the decomposition temperature for BPEA was more than 397 °C, which demonstrated that the sample was thermally stable enough for luminescence applications, since LEDs work usually at a temperature below 100 °C [26]. Furthermore, the thermal transition was scrutinized by DSC measurement (Figure S6). In the first heating, two obvious endothermic peaks were observed at 80.4 and 115.9 °C, respectively. The peak at 80.4 °C may originate from the phase transition of liquid crystals, and the peak at 115.9 °C is the melting point. During the second heating, the amorphous ground material undergoes an exothermic recrystallization process at about 57.9 °C; the peak at 106.2 °C is the melting point. Unfortunately, BPEA did not show an obvious glass-transition temperature (*T_g_*). 

### 3.2. UV–vis Absorption Spectrum

**Figure 2:**
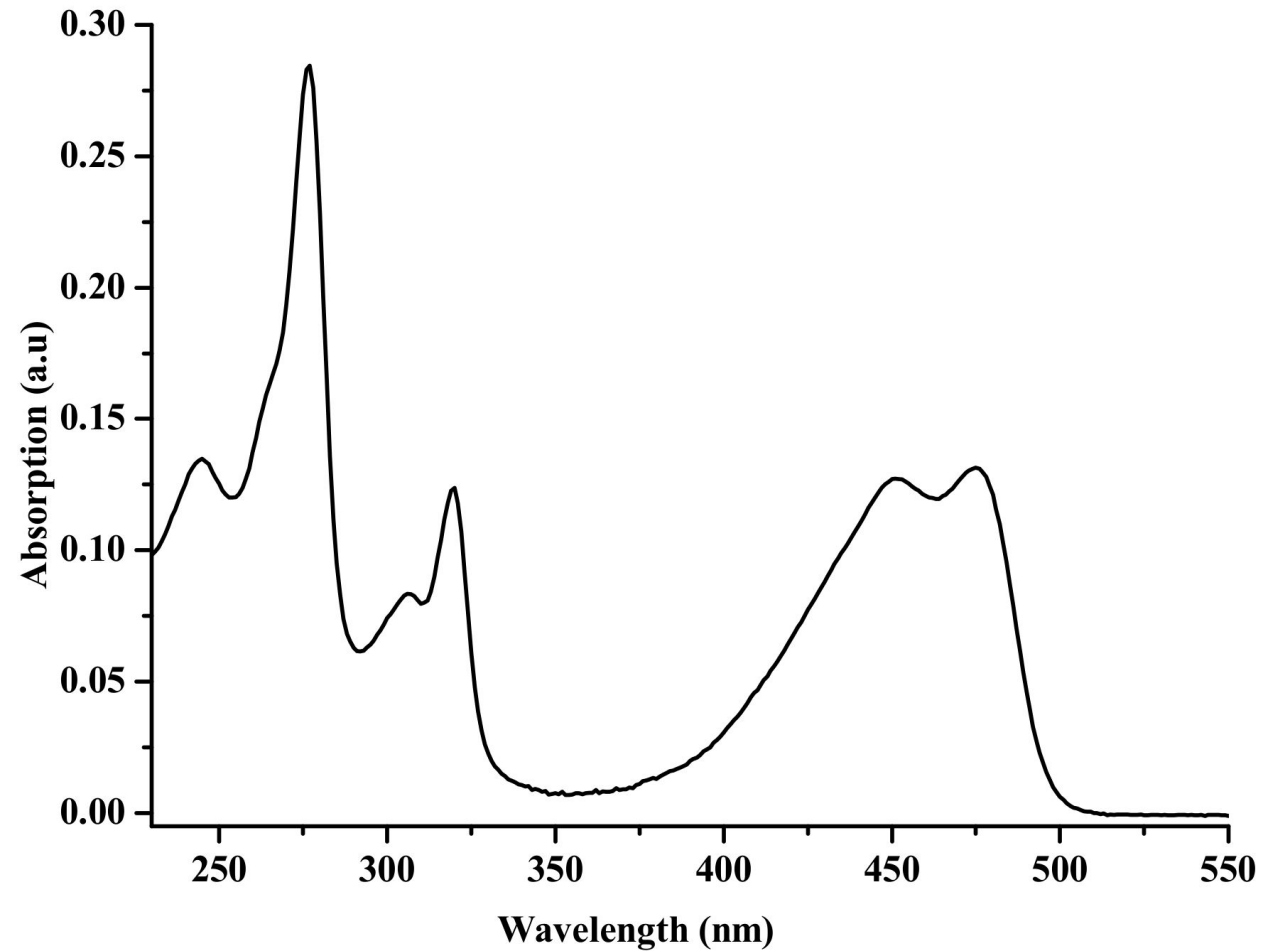
UV-vis absorption spectrum of BPEA in CH_2_Cl_2_ (3.66 × 10^−6^ mol/L).

Figure 2 shows the UV–vis absorption spectrum of BPEA in CH_2_Cl_2_ (3.66 × 10^−6^ mol/L) at room temperature. As shown, there were wide vibronic absorption bands in the region of 240–330 nm and 400–500 nm; however, the absorption band from 400 to 500 nm only originates from the π-π^∗^ transition. The strong absorption peaks are located at 243, 276, 319, 449, and 475 nm, respectively. The absorption maximum of BPEA has been extended to the visible region, which is favorable to be excited by visible-light. The molar absorption coefficient of BPEA at 475 nm is 3.55 × 10^4^ L·mol^−1^·cm^−1^.

### 3.3. Photoluminescence Properties

**Figure 3:**
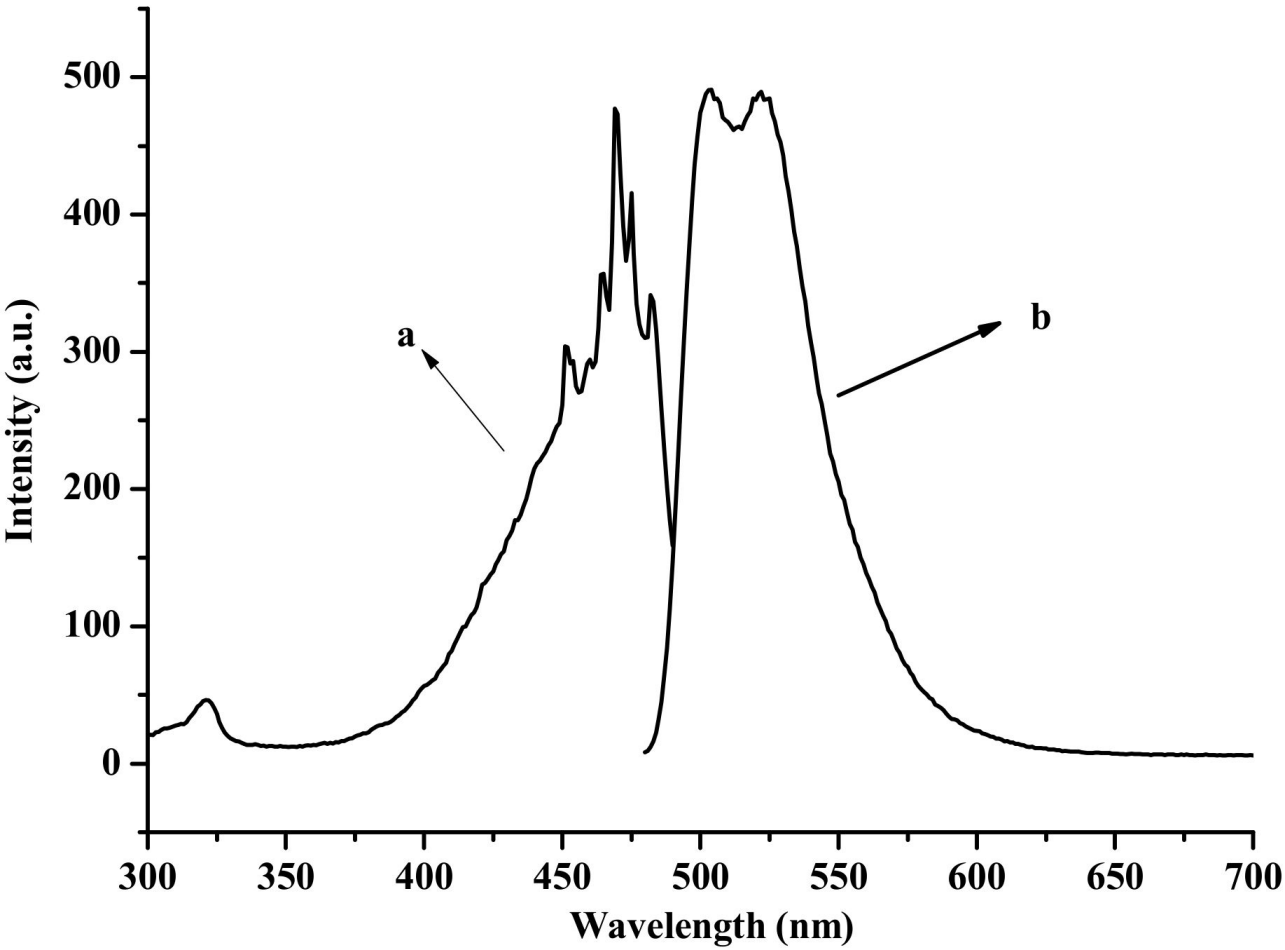
Excitation (**a**: *λ*_em_ = 503 nm) and emission spectra (**b**: *λ*_ex_ = 469 nm) of BPEA in CH_2_Cl_2_ (3.66 × 10^−6^ mol/L).

**Figure 4:**
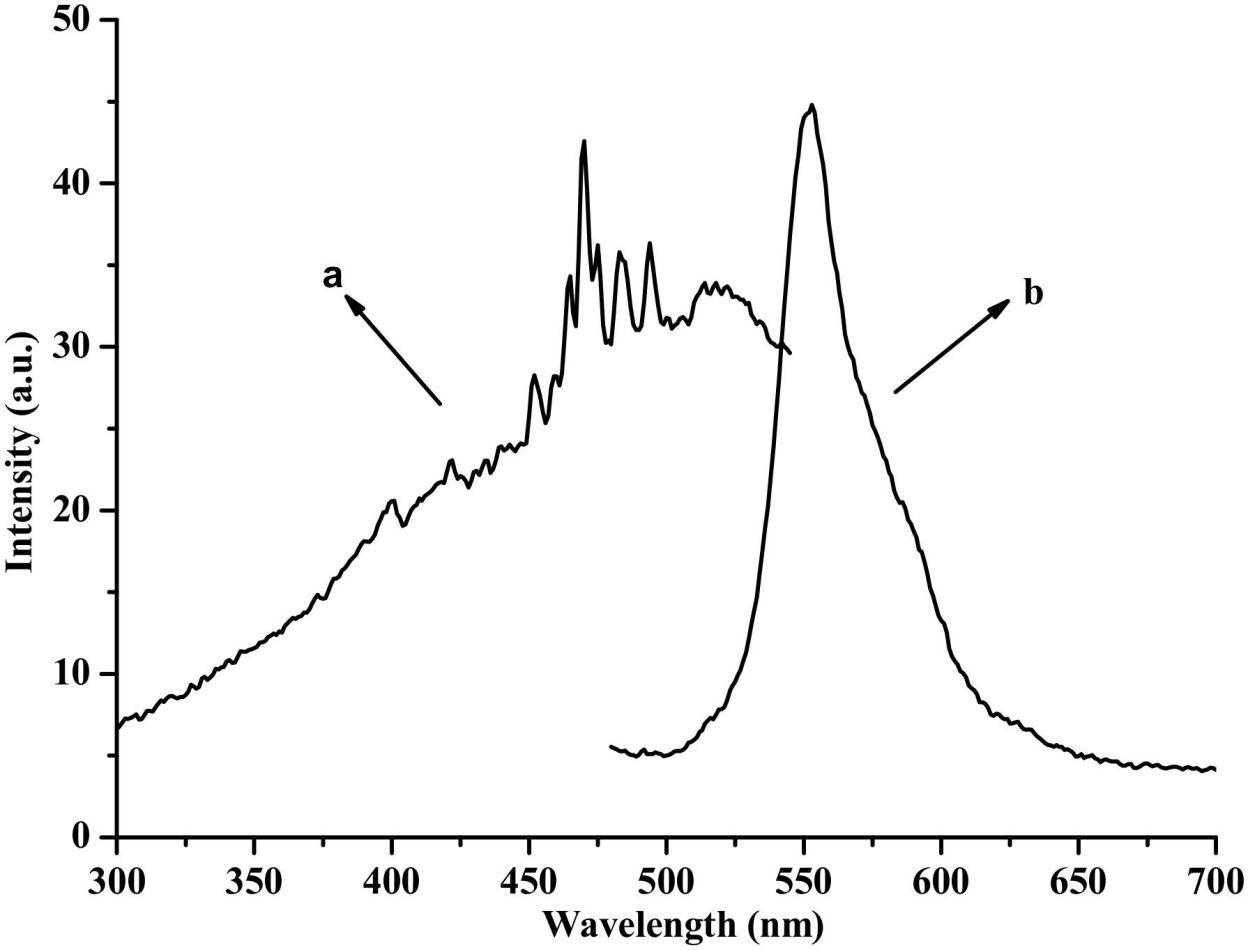
Excitation (**a**: *λ*_em_ = 554 nm) and emission spectra (**b**: *λ*_ex_ = 469 nm) of BPEA in solid state.

The excitation and emission spectra of BPEA were recorded in solution (Figure 3) and in the solid state at room temperature (Figure 4). In CH_2_Cl_2_ solution, the *λ*_max_ of excitation profile was 469 nm, and the *λ*_max_ of emission profile was 503 nm. When the dichloromethane solution of BPEA was excited by a UV lamp (365 nm), it emited a strong green light (Figure S7). The quantum yield of BPEA in pure dichloromethane was measured at room temperature using an integrated sphere system (3.70 × 10^−7^ mol/L, *λ*_ex_ = 473 nm), which can be as high as 0.938. In the solid state, the *λ*_max_ of excitation profile was 469 nm, and the *λ*_max_ of emission profile was 554 nm. The organic phosphor excited by blue light produced an intense green emission. The excitation band was extended into the visible region because of the large π-conjugation originating from the straight-backbone structure. The result would make allow the organic phosphor to avoid UV irradiation-induced decomposition in photoluminescence applications. The quantum yield of BPEA in its solid state was measured at room temperature using an integrated sphere system, based on the method described by Lin et al. [28], which is only 0.265. The quantum yield in the solid state is much lower than that in solution. As anthracene is a classical aggregation-caused quenching (ACQ) luminophore, the quantum yield in the solid state could be increased by attaching some propeller-like molecules such as silole and tetraphenylethene (TPE) moieties into the luminorphores [29]. 

### 3.4. Emission Spectra of the Fabricated LED

**Figure 5:**
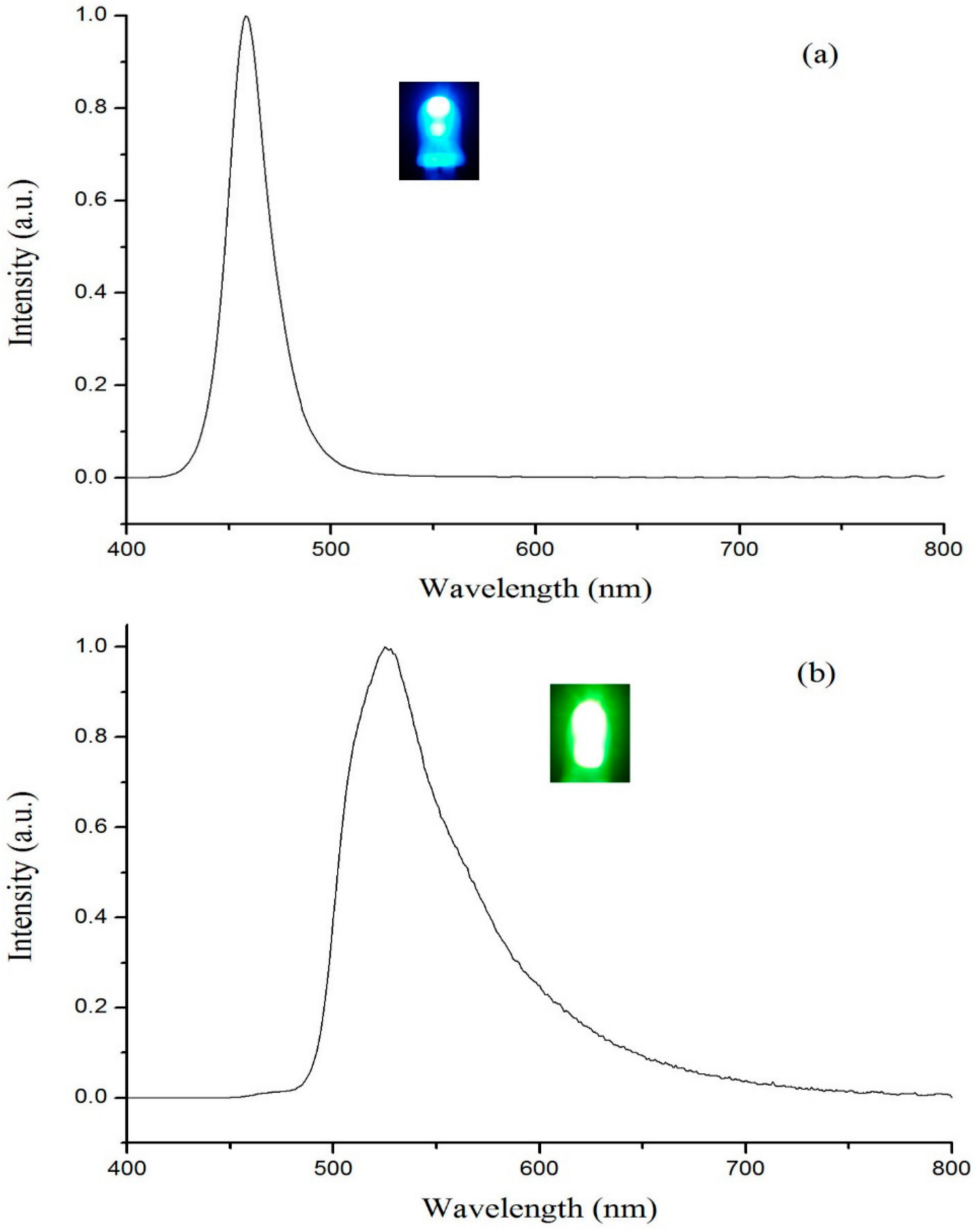
Emission spectra of the original ~460 nm-emitting InGaN LED without phosphor (**a**) and the LED with BPEA as phosphors (**b**) under excitation of 20 mA forward bias (insert: Photographs of the lighting LEDs).

BPEA was used as phosphors to fabricate LEDs in a mass ratio of 1:20 of phosphor to silicone gel with ~460 nm-emitting InGaN chips. As a comparison, most inorganic phosphors are applied in a mass ratio of 1:1 or 1:2 for the fabrication of LEDs [26,30,31]. The emission spectra and photographs of the original ~460 nm LED without phosphor (a), as well as the LED fabricated with the phosphor and an ~460 nm chip (b) under 20 mA forward bias, are shown in Figure 5. A bright green light from the LED was observed; its CIE chromaticity coordinates were calculated to be *x* = 0.3288 and *y* = 0.6199, based on its emission spectrum. The efficiency of the original LED without phosphor was only 0.39 lm/w; however, the efficiency of the fabricated LED with BPEA achieved 18.22 lm/w (Figure S8). It is should be noted that the lm/W used here is the luminous efficacy of the optical radiation. The emission at around 460 nm from the InGaN chips was completely absorbed in the spectrum of the LED with BPEA, indicating that the organic phosphor can be efficiently excited by blue light from the ~460 nm-emitting InGaN chips. These results indicate that BPEA is a good candidate for a green component in the fabrication of white LEDs with a high color-rendering index.

## 4. Conclusions

BPEA was synthesized by Sonogashira coupling reactions, and its optical properties, such as UV–vis absorption spectrum, PL, and quantum yield in solution and in a solid state, were measured. An expanded π-conjugation system in BPEA made the excitation band extend into the visible region, which exhibited intense green emission under blue-light excitation. A bright green-emitting LED (18.22 lm/w) was fabricated by coating BPEA as phosphors onto a ~460 nm-emitting InGaN chip, whose CIE chromaticity coordinates were *x* = 0.3288 and *y* = 0.6199. All the results indicate that BPEA, with high thermal stability, is a useful green-emitting material excited by blue light. This study provides a new field of applications for this kind of anthracene-acetylene compounds. 

## Supplementary Materials

The following are available online at https://www.mdpi.com/2072-666X/10/10/703/s1, Figure S1: ^1^H NMR spectrum of BPEA (500 MHz, CDCl3); Figure S2: ^13^C NMR spectrum of BPEA (125 MHz, CDCl3); Figure S3: Magnified ^13^C NMR spectrum of BPEA (125 MHz, CDCl3); Figure S4: EI-MS spectrum of BPEA; Figure S5: IR spectrum of BPEA; Figure S6: DSC profile of BPEA; Figure S7: Fluorescence emission of BPEA in CH_2_Cl_2_ (3.70 × 10^−7^ mol/L) (**a**: *λ*ex = 254 nm; **b**: *λ*ex = 365 nm); Figure S8: Emission spectrum of the fabricated LED with BPEA as phosphors.
