# Supplementary material for: Green Phosphors Based on 9,10-bis((4-((3,7-dimethyloctyl)oxy) phenyl) ethynyl) Anthracene for LED
Source: Micromachines (Basel). 2019 Oct 15;10(10):703. doi: 10.3390/mi10100703 (PMC6843851; doi:10.3390/mi10100703)
Supplement: Supplementary file 1 [file micromachines-10-00703-s001.pdf]

Supplementary Materials

Green phosphors based on 9,10-bis((4-((3,7-dimethyloctyl) oxy) phenyl) ethynyl) anthracene for LED

Xuefeng Ren [1,2], Hai Song [1,2], Jing Xiao [3,4], Hui-Juan Yu [3,4,*], Chi-Fang Peng[5] and Guang Shao [3,4,*]

1 College of Chemistry and Chemical Engineering, Hexi University, Zhangye 734000, China
2 Key Laboratory of Hexi Corridor Resources Utilization of Gansu, Hexi University, Zhangye 734000, China
3 School of Chemistry, Sun Yat-Sen University, Guangzhou 510275, China
4 Shenzhen Research Institute, Sun Yat-sen University, Shenzhen 518057, China
5 School of Food Science and Technology, Jiangnan University, Wuxi 214122, China
* Correspondence: yhjuan@mail.sysu.edu.cn (H.-J.Y.); shaog@mail.sysu.edu.cn (G.S.); Tel.: +86-20-84115178

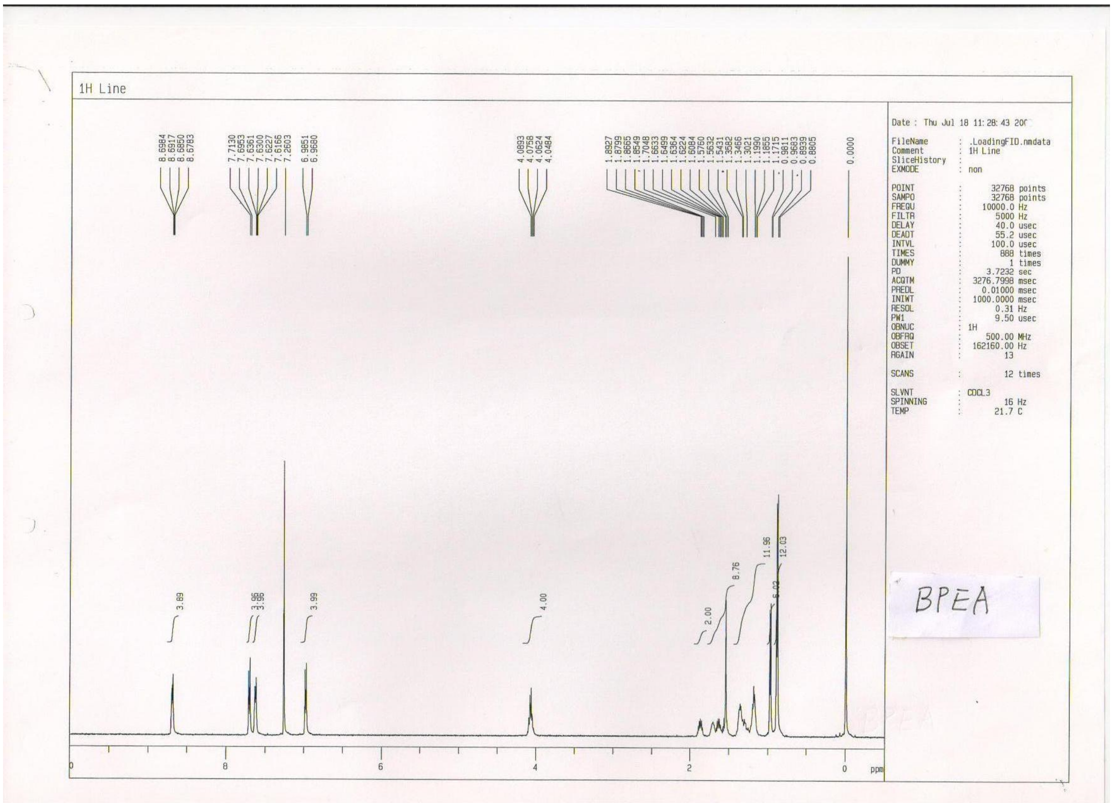

Figure S1 ^{1}H NMR spectrum of **BPEA** (500 MHz, $CDCl_3$).

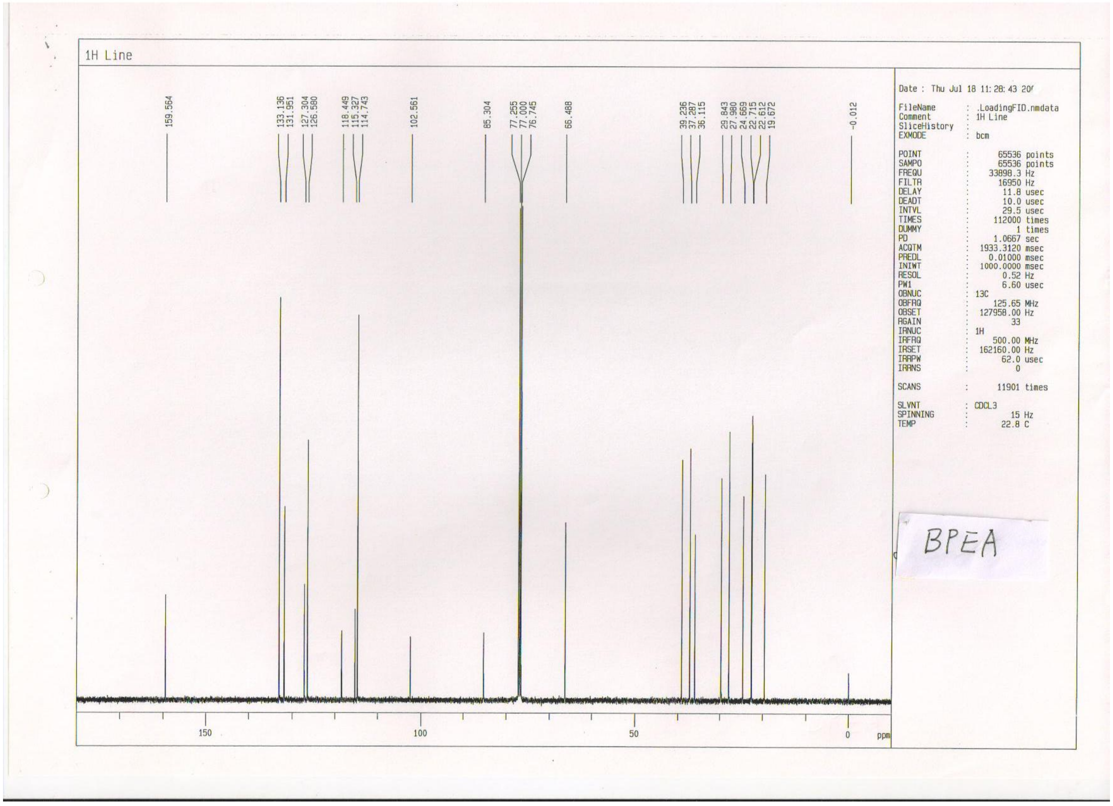

Figure S2 ^{13}C NMR spectrum of **BPEA** (125 MHz, $CDCl_3$).

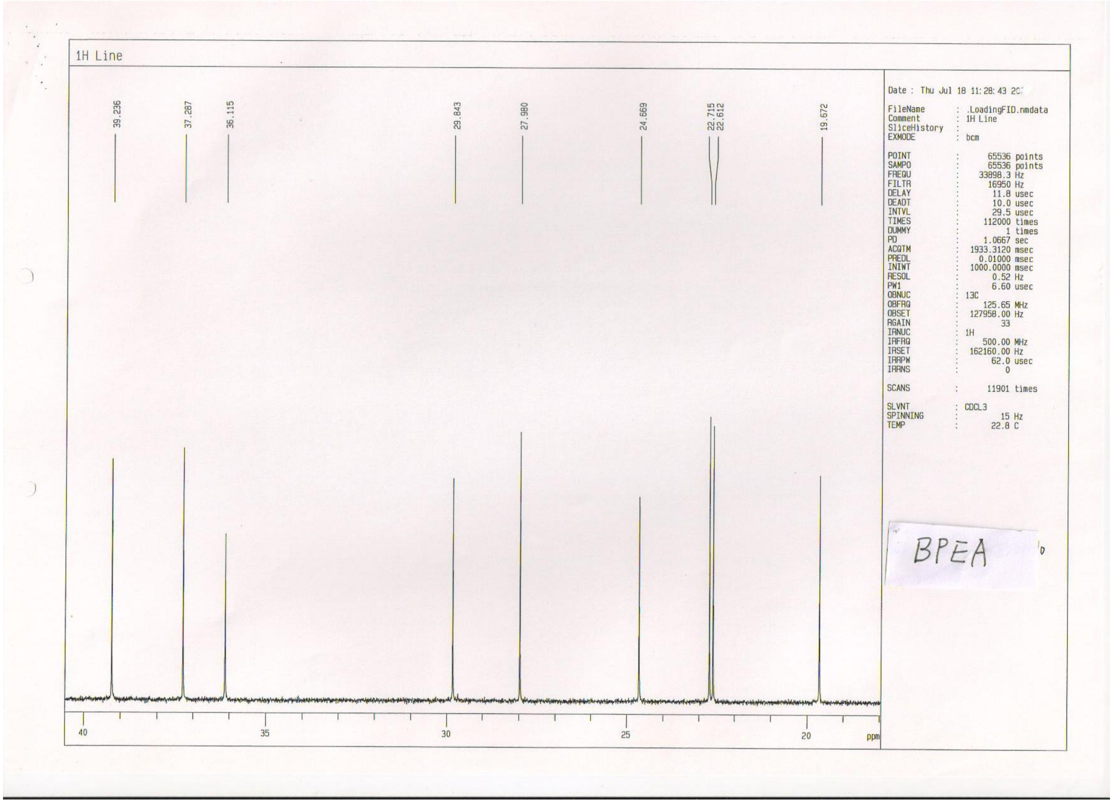

Figure S3. Magnified ^{13}C NMR spectrum of **BPEA** (125 MHz, $CDCl_3$).

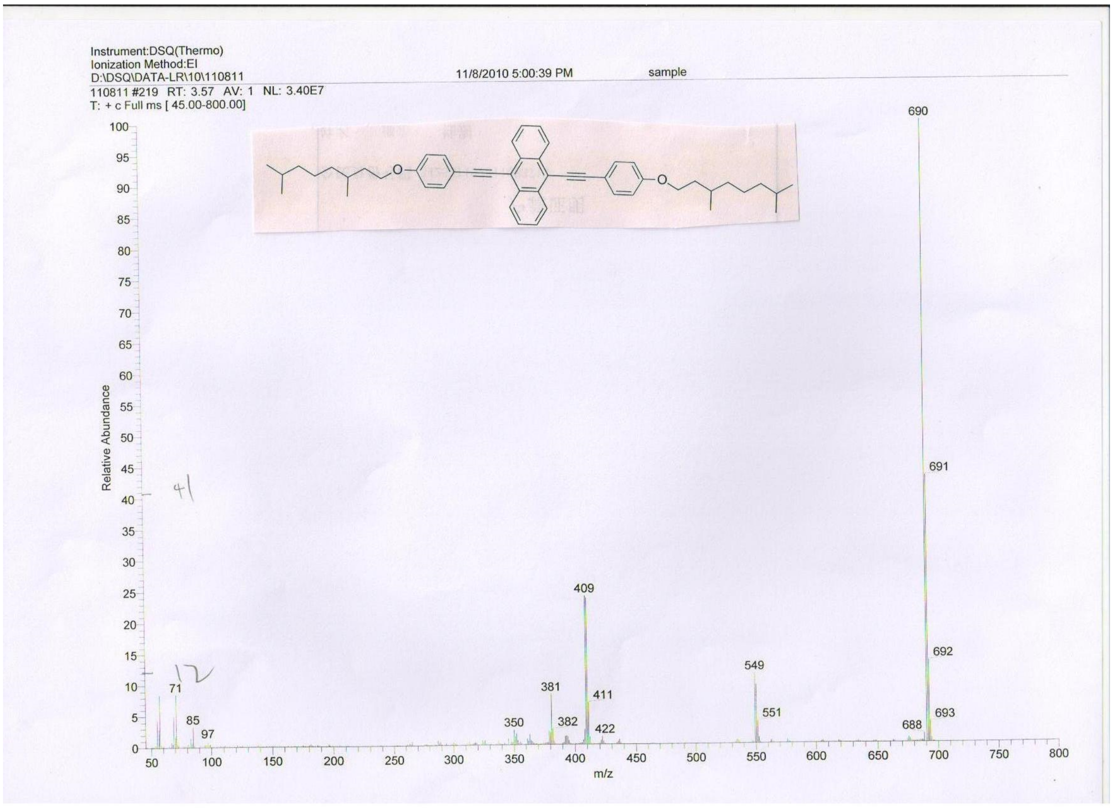

Figure S4 EI-MS spectrum of **BPEA.**

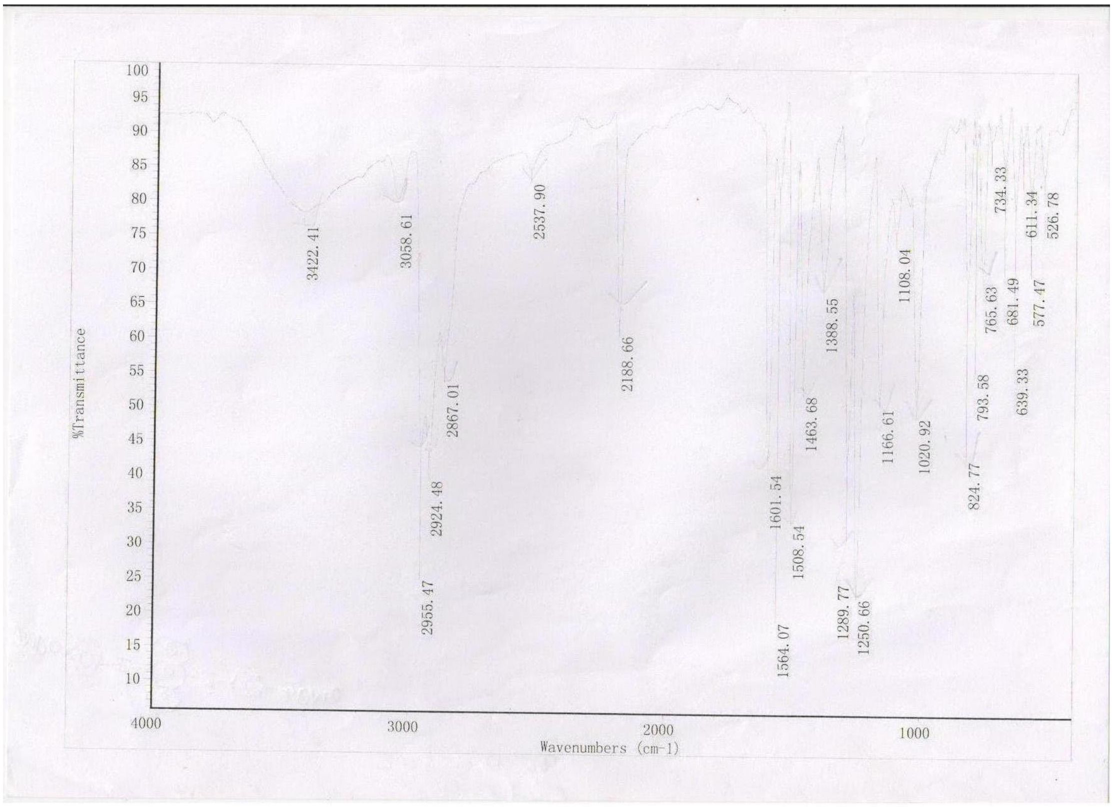

Figure S5 IR spectrum of **BPEA.**

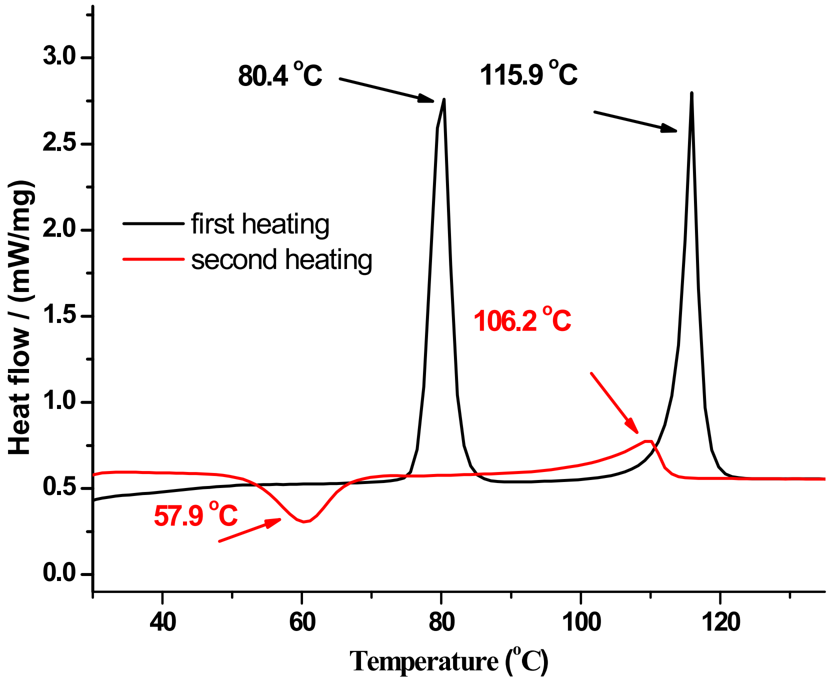

Figure S6 DSC profile of **BPEA**.

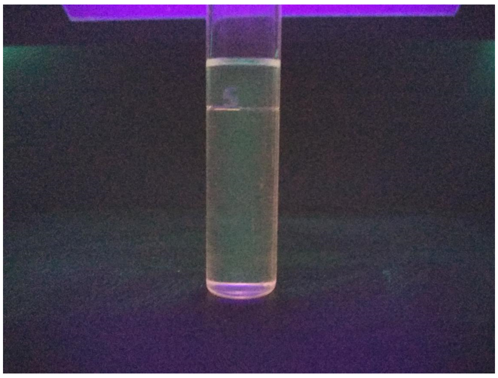

(**a**)

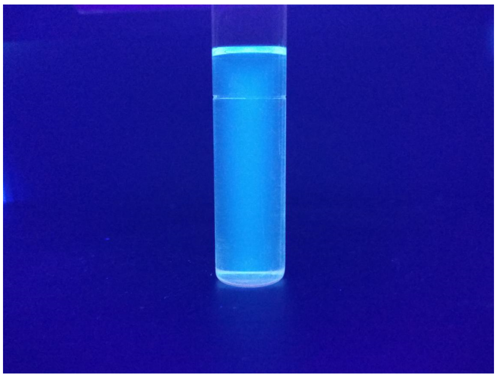

(**b**)

Figure S7 Fluorescence emission of BPEA in CH_2Cl_2 (3.70×10^{-7} mol/L) (**a**: λ_{ex} = 254 nm; **b**: λ_{ex} = 365 nm).

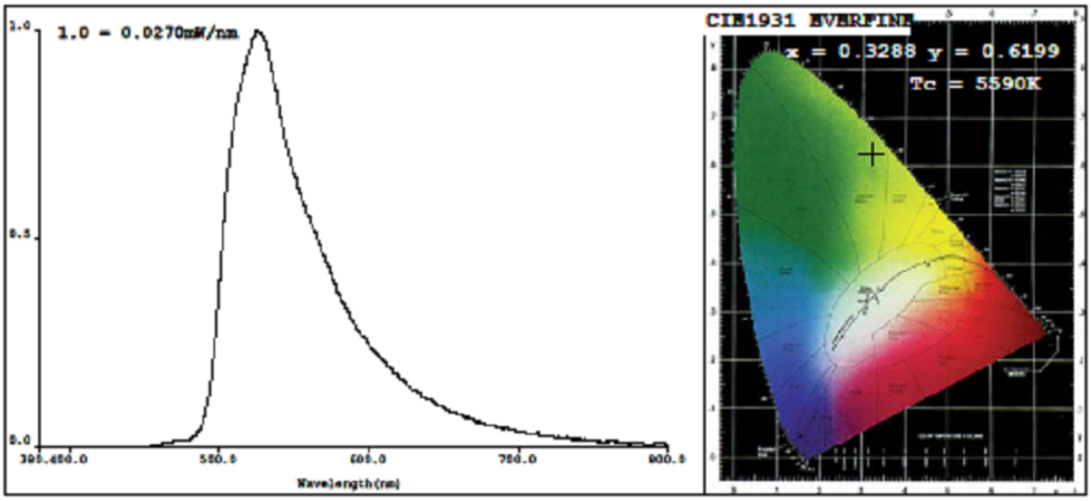

Figure S8 Emission spectrum of the fabricated LED with **BPEA** as phosphors.
